# Radiotherapy for Refractory Cystic Progression in Basal Ganglia Pilocytic Astrocytoma: A Case Report

**DOI:** 10.7759/cureus.104207

**Published:** 2026-02-24

**Authors:** Shohei Nagasaka, Kohei Suzuki, Takeshi Saito, Yoshiteru Nakano, Junkoh Yamamoto

**Affiliations:** 1 Neurosurgery, University of Occupational and Environmental Health, Kitakyushu, JPN

**Keywords:** cystoperitoneal shunt, cyst recurrence, endoscopic cyst fenestration, pilocytic astrocytoma (pa), radiotherapy (rt), ventriculoperitoneal (vp) shunt

## Abstract

Pilocytic astrocytoma (PA) is a common low-grade pediatric glioma that often exhibits cystic components. Although cyst-related symptoms are usually controlled by gross-total resection or cerebrospinal fluid (CSF) diversion procedures, management can be challenging when tumors arise in deep or eloquent locations and are associated with highly proteinaceous cyst fluid. We report a case of a 14-year-old girl with PA arising in the basal ganglia who developed recurrent and refractory cystic enlargement following chemotherapy. Despite multiple interventions, including Ommaya reservoir placement, repeated cyst aspirations, endoscopic cyst fenestrations, and cystoperitoneal and ventriculoperitoneal shunts, cyst control was repeatedly compromised due to obstruction associated with extremely high cyst fluid protein levels. Although cyst-ventricle communication resulted in a marked reduction in protein concentration, recurrent obstruction and cyst re-expansion persisted. Given the refractory nature of the cystic lesion and the absence of endocrine dysfunction, fractionated radiotherapy was selected. Following radiotherapy (60 Gy in 30 fractions), magnetic resonance imaging revealed reduced contrast enhancement and stabilization of the cystic component. The patient remained clinically stable without treatment-related adverse events or cyst enlargement during the two-year follow-up. This case highlights the limitations of conventional surgical and CSF diversion strategies for cystic PA with high proteinaceous content. Radiotherapy may be a viable therapeutic option for refractory cystic progression in patients with PA, even in the absence of solid tumor growth.

## Introduction

Pilocytic astrocytoma (PA) is classified as a central nervous system (CNS) World Health Organization (WHO) grade 1 tumor. It accounts for approximately 5.1% of all gliomas and is most commonly diagnosed in children [1]. Clinical manifestations are often related to ventricular obstruction and elevated intracranial pressure, and may include headache, nausea, vomiting, cranial nerve dysfunction, focal seizures, and hemiparesis [2]. The cerebellum was the most commonly involved site (40%), followed by the supratentorial regions (35%), optic pathway/hypothalamus (11%), and brainstem (9%) [3]. Occurrence in deep gray matter structures such as the basal ganglia is uncommon and poses unique therapeutic challenges.

Radiologically, PAs usually present as well-circumscribed solid, cystic, or mixed lesions. Computed tomography (CT) reveals round or oval iso- to hypointense masses with strong contrast enhancement, whereas magnetic resonance imaging (MRI) typically shows iso- to hypointensity on T1-weighted images and hyperintensity on T2-weighted images and fluid-attenuated inversion recovery sequences, accompanied by intense enhancement [4,5].

Management of PA generally consists of surgical resection, followed by observation, chemotherapy, or radiotherapy for residual tumors [6]. Surgical resection, especially gross-total resection (GTR), is widely recognized to be associated with improved progression-free and overall survival, as well as reduced risks of progression and recurrence, and is most commonly achieved in cerebellar tumors [7,8]. Overall, PAs have an excellent prognosis, with reported 10-year survival rates exceeding 90% [3]. However, approximately 20% of tumors arise in deep or critical locations, such as the brainstem or hypothalamus, where complete resection is not feasible [9,10]. In such cases, biopsy followed by adjuvant therapies, most commonly chemotherapy, is considered. Postoperative radiotherapy may also be employed in selected patients [10,11].

 PAs often contain cystic components that may contribute substantially to mass effect and neurological deterioration. In such cases, cyst-related symptoms are controlled with surgical intervention, ventriculoperitoneal (VP) shunt, radiation therapy, or chemotherapy [12]. However, some cystic lesions demonstrate persistent or recurrent enlargement despite these measures, a condition referred to in this report as refractory cystic progression. Radiotherapy is generally reserved for progressive solid tumor components in PA, and its role in isolated cystic progression remains poorly defined.

Here, we report a case of basal ganglia PA with refractory cystic enlargement caused by extremely high cyst fluid protein content, in which multiple surgical and cerebrospinal fluid (CSF) diversion strategies failed. Fractionated radiotherapy resulted in a two-year stabilization of the cystic component. This case highlights a potential role for radiotherapy as a salvage option for refractory cystic PA.

## Case presentation

**Figure 1:**
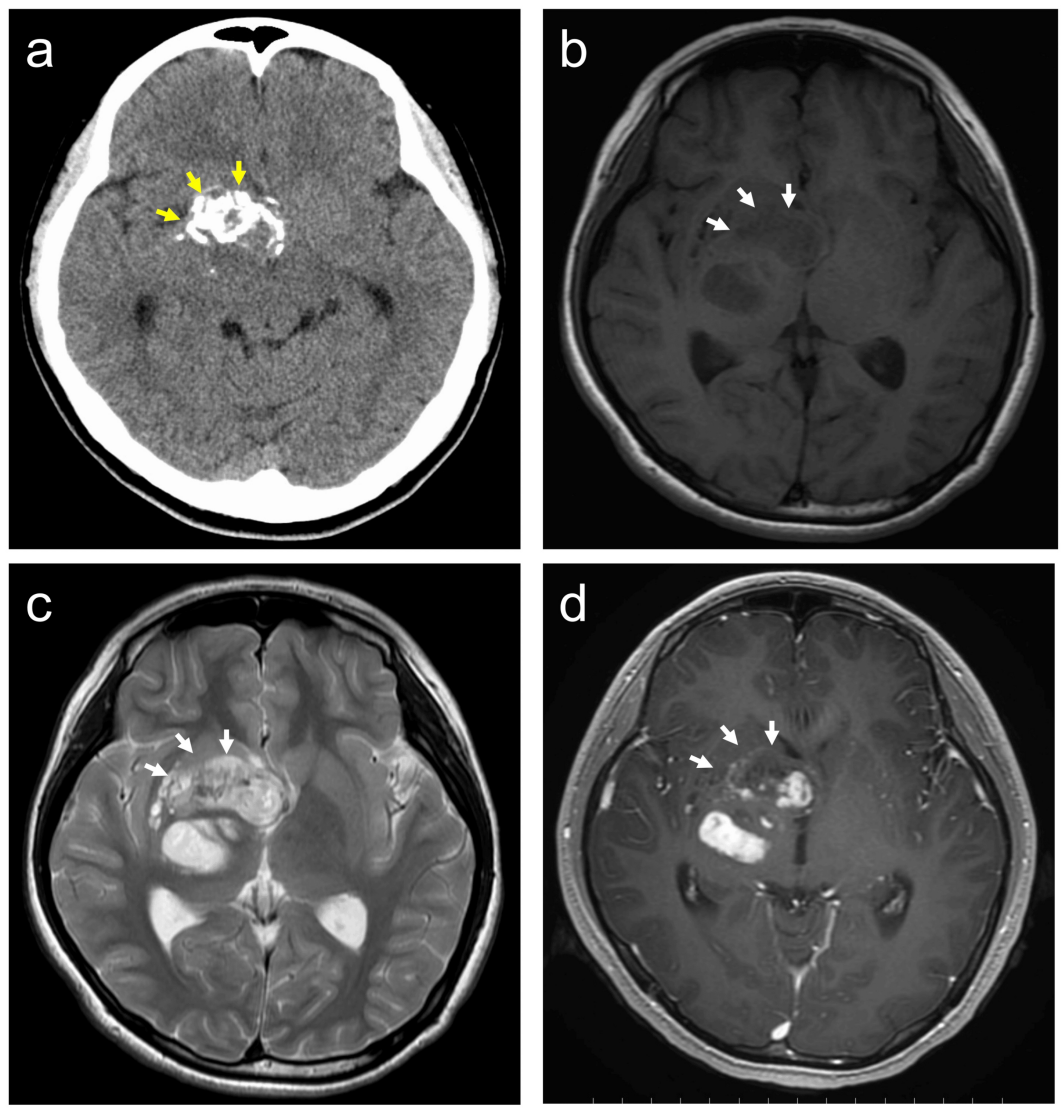
Initial radiological findings. (a) Intratumoral calcification (yellow arrows) on computed tomography. (b,c) Magnetic resonance imaging (MRI) revealed hypointensity on T1-weighted images (WI) and heterogeneous hyperintensity on T2-WI of the tumor (arrows). (d) Contrast-enhanced T1-WI showing heterogeneous enhancement within the solid component of the tumor (arrows).

A 14-year-old girl with no significant past medical history and an unremarkable family history presented with progressive left-sided weakness. Neurological examination revealed left hemiparesis without cranial nerve deficits or endocrine abnormalities. She had no history of seizures. There were no clinical features suggestive of neurofibromatosis type 1. Brain MRI and CT revealed a solid-cystic mass with intratumoral calcification located in the right basal ganglia (Figure 1A-1D).

**Figure 2:**
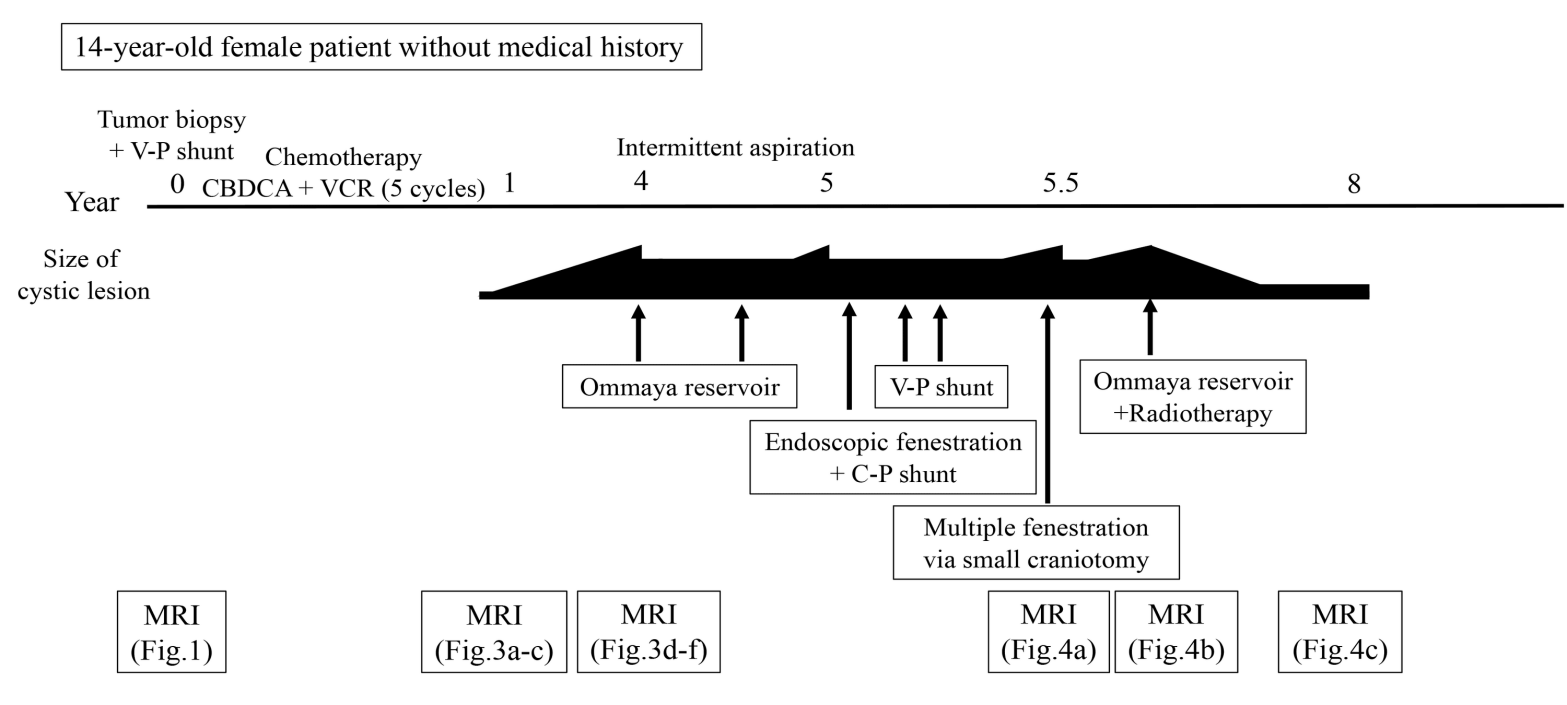
Timeline of the patient’s clinical course. Chronological summary of treatments and disease progression, including biopsy, chemotherapy, Ommaya reservoir placement, cyst fenestrations, shunt procedures, and radiotherapy. Abbreviations: CBDCA, carboplatin; VCR, vincristine; V-P, ventriculo-peritoneal; C-P, cysto-peritoneal; MRI, magnetic resonance imaging

She underwent a tumor biopsy via a transventricular approach at another hospital, which confirmed PA diagnosis (CNS WHO grade 1) (Figure 2).

**Figure 3:**
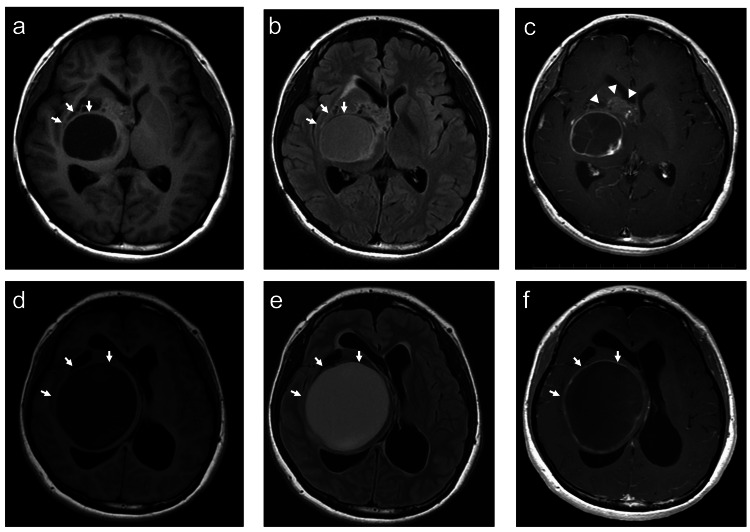
Response to chemotherapy and subsequent progression (a,b) After chemotherapy, MRI revealed hypointensity on T1-WI and isointensity on fluid-attenuated inversion recovery (FLAIR) within the cystic component (arrows). (c) The contrast-enhancing solid tumor component shows interval regression on contrast-enhanced T1-WI (arrowheads). (d,e) Follow-up MRI obtained two years after chemotherapy demonstrates progressive enlargement of the cystic component, with hypointensity on T1-WI and hyperintensity on FLAIR (arrows). (f) Contrast-enhanced T1-WI revealed enhancement of the cyst wall (arrows).

Postoperatively, a V-P shunt was placed to manage secondary hydrocephalus. The patient was subsequently referred to our hospital for treatment. She received five cycles of chemotherapy comprising carboplatin and vincristine. Follow-up MRI demonstrated tumor regression with associated cyst formation (Figure 3A-3C). During outpatient follow-up, the cystic component gradually enlarged over three years (Figure 3D-3E).

**Figure 4:**
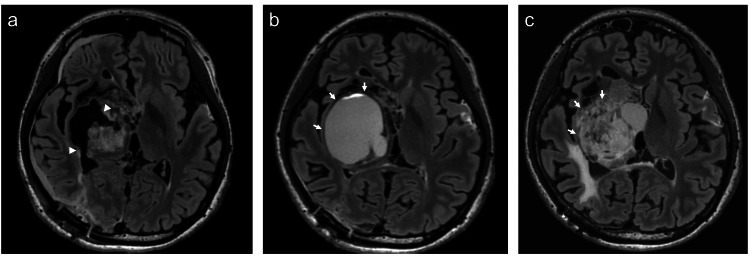
Radiological course following fenestration and radiotherapy (a) Postoperative FLAIR obtained after multiple cyst fenestrations (arrowheads) were performed via a small craniotomy. (b) Follow-up FLAIR one month after surgery demonstrates progressive enlargement of the cystic component despite fenestration (arrows). (c) FLAIR obtained two years after radiotherapy showed stabilization of the cystic component without further enlargement (arrows).

Because of progressive cyst enlargement and worsening left hemiparesis, an Ommaya reservoir was placed for cyst decompression. Weekly cyst aspirations were required to achieve symptomatic relief. The aspirated fluid was yellowish and demonstrated an extremely high protein concentration of 5,100 mg/dL. Although cyst volume was temporarily reduced, the reservoir catheter became occluded and required replacement because of the highly proteinaceous content. One year after initiation of reservoir management, the patient’s clinical status deteriorated significantly, with worsening left-sided hemiparesis and severe headaches. MRI demonstrated marked expansion of the cystic component due to reservoir obstruction. Endoscopic cyst fenestration was performed, and a cystoperitoneal (C-P) shunt was placed via the right posterior horn. However, this shunt subsequently became obstructed, prompting repeat endoscopic multiple cyst fenestrations and placement of a right posterior horn V-P shunt. Moreover, owing to the subsequent obstruction of the catheter, an additional V-P shunt was placed via the left anterior horn, after which the patient was discharged. One month after the additional shunt replacement, follow-up MRI demonstrated re-enlargement of the cystic component. Fenestration of the cyst was performed through a small craniotomy via the right posterior horn (Figure 4a). Histopathological examination confirmed the diagnosis of PA, and molecular analysis was negative for the BRAF V600E mutation. Despite these repeated surgical interventions, obstruction of the fenestration site recurred within two months, and progressive cyst enlargement was observed on follow-up MRI (Figure 4b). Given the refractory nature of the cystic lesion and repeated failure of surgical and CSF diversion procedures, further treatment options were carefully discussed. Because there was no evidence of endocrine dysfunction or progressive solid tumor growth, an Ommaya reservoir was reinserted for temporary cyst decompression followed by fractionated radiotherapy. The patient received a total dose of 60 Gy in 30 fractions. Post-radiotherapy MRI demonstrated a reduction in contrast enhancement and stabilization of the cystic component. At the two-year follow-up, the patient remained clinically stable. Her neurological deficits, including left hemiparesis, were unchanged, and no adverse events were observed (Figure 4c).

## Discussion

PA is a CNS WHO grade 1 tumor with generally favorable long-term outcomes [3]. GTR is strongly associated with prolonged progression-free and overall survival; however, GTR is often difficult to achieve for tumors arising in deep or eloquent locations [8]. Past reports have shown that approximately 20% of PAs occur in anatomically challenging regions, including the brainstem, hypothalamus, optic pathway, and basal ganglia, where surgical morbidity must be carefully balanced against oncological control [3,8].

Cystic components are frequently observed in PA and can substantially contribute to mass effect and neurological deterioration [12]. Standard approaches for cyst management include Ommaya reservoir placement, cyst fenestration, and CSF diversion procedures [12,13]. These strategies are generally effective for decompression; however, their durability depends on long-term catheter patency and maintenance of cyst-CSF communication. In the present case, extremely high cyst fluid protein content led to repeated obstruction of catheters and shunt systems, resulting in rapid cyst enlargement despite multiple surgical interventions. In addition, the small size of the fenestration may have contributed to obstruction.

Molecularly targeted therapy represents an additional treatment option for selected patients with PA. BRAFV600E mutations have been reported in approximately 16% of PAs [2,14], and BRAF inhibitors such as dabrafenib have demonstrated efficacy in this molecular subgroup [2,15]. However, our patient was negative for the BRAFV600E mutation, limiting the applicability of targeted therapy and necessitating consideration of alternative strategies.

Radiotherapy is typically reserved for progressive or residual solid tumor components of PA following surgery and/or chemotherapy [8,12]. Several reports have demonstrated acceptable tumor control and survival with radiotherapy in unresectable or progressive PA [3,8]. In contrast, reports describing radiotherapy for isolated cystic progression without solid tumor growth are extremely limited. In our patient, fractionated radiotherapy resulted in stabilization of the cystic component and clinical stability for two years, despite repeated failure of surgical and CSF diversion procedures.

This case illustrates a rare but challenging presentation of PA characterized by refractory cystic progression driven by extremely high cyst fluid protein content. Our findings suggest that radiotherapy may represent a salvage therapeutic option for selected patients with cystic PA when conventional surgical and CSF diversion strategies are ineffective.

This report has several limitations. First, this is a single case observation, and the generalizability of the findings is therefore limited. Second, serial quantitative measurements of cyst volume and solid tumor volume were not available, which precluded formal volumetric analysis. Third, standardized radiological response criteria and toxicity grading systems were not uniformly applied. Finally, the biological mechanism by which radiotherapy stabilized the cystic component remains speculative.

## Conclusions

Recurrent cystic progression associated with PA can be particularly difficult to manage when cyst fluid protein concentrations are markedly elevated, leading to repeated failures of surgical and CSF diversion strategies. This case suggests that radiotherapy may be an effective treatment option for refractory cystic lesions in PA even when progression is limited to the cystic component without solid tumor growth.
